# Evaluation of a dementia awareness game for health professions students in Northern Ireland: a pre-/post-test study

**DOI:** 10.1186/s12909-024-05656-z

**Published:** 2024-06-18

**Authors:** Stephanie Craig, Heather E. Barry, Gillian Carter, Patrick Stark, Gary Mitchell, Sonya Clarke, Christine Brown Wilson

**Affiliations:** 1 School of Nursing and Midwifery, Queen’s University Belfast, Belfast, Northern Ireland; 2 School of Pharmacy, Queen’s University Belfast, Belfast, Northern Ireland

**Keywords:** Dementia, Nurse education, Pharmacy education, Serious games, Students, Pedagogical research, Constructivist pedagogy, Quantitative research methods

## Abstract

**Background:**

Dementia is a prevalent global health issue, necessitating comprehensive education for healthcare practitioners and students. Nursing and pharmacy students, provide support across healthcare settings often working as frontline caregivers. Therefore, it is imperative to equip these students with a profound understanding of dementia. The aim of this study was to evaluate whether a serious dementia game co-designed with stakeholders, students, and people living with dementia improved the attitudes of nursing and pharmacy students.

**Methods:**

A pretest-posttest design was used to assess the attitudes of health professions students (nursing and pharmacy) towards dementia. The Approaches to Dementia Questionnaire (ADQ) was administered before and after playing a serious Dementia Game. The ADQ measured the total score, Hope subscale, and Recognition of Personhood subscale. Matched pairs t-test was used for analysis conducted with IBM SPSS statistics 27.

**Results:**

A diverse cohort of 505 participants from one university in Northern Ireland participated, with 461 matched pairs used for analysis. Both nursing and pharmacy students demonstrated a significant increase in overall dementia attitudes post-gameplay, with nursing students showing an increase from 79.69 to 83.59 and pharmacy students from 75.55 to 79.86. Subscales for Hope (Nursing = 28.77 to 31.22, Pharmacy = 26.65 to 29.20). and Recognition of Personhood also exhibited significant improvement (Nursing = 50.93 to 52.38, Pharmacy = 48.89 to 50.67). Demographic data revealed predominantly female participants, a lack of personal connections to dementia, and varied training experiences.

**Discussion:**

The study highlights the efficacy of the serious Dementia Game in enhancing attitudes to dementia amongst health professions students, indicating its potential as an educational tool. The study contributes to the growing body of evidence supporting serious games and gamification in healthcare education.

## Background

Throughout primary, secondary, and tertiary healthcare settings, healthcare professionals play a crucial role in providing comprehensive support [1] to those with dementia, from the initial stages of pre-diagnosis to end-of-life care [2]. Each year, over 10 million new cases of dementia are documented, with more than 55 million individuals affected globally [3]. Nurses and pharmacists are integral members of the healthcare workforce worldwide [4–7]. Consequently, individuals receiving a dementia diagnosis often receive assistance from both student nurses and student pharmacists, who represent the future of healthcare [8, 9]. To prepare for their clinical placements, these students require a solid foundation in dementia education to effectively provide care and support to individuals living with dementia [10].

Within higher education institutions, dementia education must be integrated into the curriculum to adequately fulfil the educational requirements and standards set forth by the Nursing and Midwifery Council (NMC) and the General Pharmaceutical Council (GPhC). Within the author’s institution, dementia is extensively integrated into the nursing and pharmacy undergraduate programs, covering various biomedical aspects of care. These include the aetiology, pathophysiology, clinical manifestations of dementia diseases, nursing and medical interventions, pharmacology, non-pharmacological treatments, and palliative care. However, aspects of patients’ emotions upon diagnosis and how to communicate effectively with people living with dementia are not always explicitly covered in both undergraduate programmes. To address this gap, the Alzheimer’s Society ‘Dementia Friends’ program has been introduced to both programmes [11, 12]. This initiative, part of a global effort, aims to raise awareness and cultivate empathy among learners, recognising the pivotal role of nurses and pharmacists in supporting individuals with dementia across various stages of care [11, 13]. Moreover, the recent COVID-19 pandemic, alongside advancements in digital technology, has led to the emergence of more digital or asynchronous learning opportunities in higher education. One innovative approach is the use of digital serious games to enhance learning experiences.

A recent systematic review and meta-analysis [14] suggests that digital games can be equally beneficial as a stand-alone or multi-component programme while appealing to a wide population regardless of age or gender. Gamification is a concept that combines gaming components with teaching to raise student engagement levels in a classroom setting [15]. A serious game helps players learn the material and hone their skills through gaming [16]. A growing number of studies have shown that using “serious games” to train health professionals can increase participation, user retention, knowledge, and cooperation [14, 17]. The use of serious games is a well-known teaching strategy in the realm of nursing education [16, 18–22] and pharmacy education [23–25]. Supporters of digital gaming also stress the games’ accessibility and practicality [26]. Serious games offer a novel method of teaching and improving comprehension; for instance, knowledge of dementia could be enhanced through the use of serious games in healthcare education [27].

This study builds upon prior research demonstrating significant enhancements in nursing student knowledge following the use of a digital game [27]. Expanding upon this foundation, our aim is to broaden the scope of evaluation to explore the potential impact of serious games on not only knowledge acquisition but also on the cultivation of positive attitudes and values towards dementia among nursing and pharmacy students. To our knowledge, no empirical studies have evaluated the effectiveness of a digital serious game to enhance undergraduate nursing and pharmacy students’ attitudes of dementia. Therefore, the aim of this project was to evaluate the impact of a dementia serious game on the attitudes of nursing and pharmacy students.

## Methods

### Intervention—the dementia game

A digital serious game called The Dementia Game [28] was co-designed through stakeholder involvement, student nurses and with people living with dementia [29]. It is a web-based application (HTML5) that operates on any device with an internet connection. Multiple-choice questions regarding dementia are provided in a random order, and players must follow a path to the finish line by answering these questions correctly. These questions were jointly developed with people living with dementia to ensure key misconceptions about dementia were addressed. Points are awarded for answering questions correctly, and additional points are awarded for finishing the game. Players can challenge others to a game and record their scores. Participants have unlimited access to playing the Dementia Game and each game takes around 90 seconds to complete. The serious game is freely accessible here: www.dementiagame.com.

### Design

A pretest-posttest design was used to determine the effectiveness of the serious digital game on a cohort of health professions students (nursing and pharmacy undergraduate students). Before and after playing the Dementia Game, health profession students’ attitudes towards dementia were assessed using the Approaches to Dementia Questionnaire (ADQ) [30].

### Participants

Potential participants were recruited by two gatekeepers who were independent from this study. The gatekeepers provided eligible participants (Table 1) with access to the serious game as part of their existing education on dementia before their first taught class on dementia. The gatekeepers also wrote to students to inform them about the evaluation for this study which involved a pre-test before playing and a post-test after playing. All participants were informed that they could play the game without undertaking the pre- or post-test. The total available sample was 624 (*n* = 544 from nursing and *n* = 80 from pharmacy). Nursing students were in Year 1 of their degree, Pharmacy students were in Year 3 of their degree. A total of *n* = 505 students responded to at least the pre-test survey (80.93% response rate) and *n* = 461 fully completed the study (73.87% response rate).

**Table 1:** Inclusion/exclusion criteria for participants

Inclusion criteria	Exclusion criteria
Students currently enrolled at Queen’s University Belfast	Students enrolled at another University in Northern Ireland
Students currently studying a nursing or pharmacy degree	Students from the Faculty of Medicine, Health and Life Science completing Medicine or Dentistry
Students who had played the dementia awareness game as part of their module learning	

### Evaluation questionnaire

The 19-item Approaches to Dementia Questionnaire (ADQ) [30] with modifications validated by Cheston et al. [31] has been demonstrated to be a valid and reliable instrument [30–32]. Each item has a five-point Likert scale measuring level of agreement/disagreement, giving a possible total score range of 19–95, with higher scores demonstrating a more positive attitude towards people living with dementia. Two further subscales measure ‘Recognition of personhood’ focusing on how a person with dementia is viewed as an individual with capabilities; and ‘Hope’ indicating either an optimistic or pessimistic approach to someone with dementia. Overall, the ADQ makes it possible to assess health professions students’ attitudes towards dementia by measuring the extent to which those playing the game acknowledge people affected by dementia as unique individuals with the same value as any other person, and it also highlights any sense of optimism or pessimism the person had about the abilities and the future of a person affected by dementia [28].

A short demographic questionnaire of seven questions was also appended to the pre-game questionnaire, including three questions related to students’ experiences of knowing someone with dementia, working with those living with dementia, and training/education received about dementia.

### Data collection

**Fig. 1:**
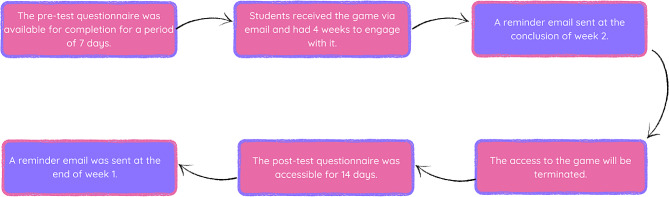
Data collection flow chart

The students who chose to participate in the study completed the pre- and post-questionnaires during a four-week period when they had access to the game. The pre-test questionnaire was the initial time point (T_0_), and participants had seven days to complete the questionnaire via a web link. Following the conclusion of the pre-test period, a weblink to the serious game was emailed to every participant, by one of the independent gatekeepers, who had completed the pre-test questionnaire and gave their online informed consent to play the game. The game was fully accessible to these participants across four weeks and there were no restrictions as to the number of times it could be played. At the midpoint of this gaming period (the end of week two), an email with instructions on how to access the game was redistributed to every participant by both gatekeepers. Access to the game was closed after four weeks. Participants were then emailed their post-test questionnaire (T_1_) to complete. Participants had access to the post-test questionnaire for 14 days, and a reminder email was issued after one week (Fig. 1). Data collection was carried out between March 2022 and May 2023.

### Ethics

This study received ethical approval by Queen’s University Belfast’s Faculty of Medicine, Health, and Life Sciences Research Ethics Committee (MHLS 22_57). Online informed consent was obtained from all questionnaire participants. All methods were performed in accordance with the Declaration of Helsinki [33].

### Consent

All eligible participants (*n* = 505) were informed via email of the study and their ability to participate by a person unrelated to the project. It was highlighted that their decision to take part in the study was voluntary and would not affect how they progressed through the course or how well they performed on a module. Students needed access to their own laptop, tablet, or phone to complete the questionnaires and play the game. Participants were unable to interact with a non-digital format of the intervention or complete hard copies of the questionnaires due to the intervention’s mode of delivery.

### Data analysis

All data were transferred from MS Forms to a MS Excel spreadsheet, where they were cleaned, coded and scored according to the ADQ guidelines. Data were quality checked by two members of the research team (PS and GC). Demographic data were reviewed using descriptive analysis. Matched pairs for the ADQ scores at baseline and follow-up were analysed using dependent t-tests, with a two-tailed significance level set at α = 0.05. Mean gain was reported for each, and a Bonferroni corrected alpha cut off to control for false positive risk with multiple significance tests (*p* < 0.008 was significant). Alpha (0.05) was divided by the number of significance tests (6) to get the value of *p* = 0.008. Analysis was conducted using IBM SPSS statistics 27 [34].

## Results

In total, 505 participants completed the pretest questionnaire and subsequently played the Dementia Game (74 pharmacy students and 431 nursing students). Of these, 461 participants then also completed the posttest questionnaire, therefore only 461 matched pairs were used for analysis (reflecting 8.71% attrition from pre-test to analysis). This missing data occurred due to the students not completing the post-test questionnaire, despite having accessed the game. Participants were not supervised during data collection, and so this attrition could not be mitigated further. Table 2 provides the demographics of these respondents in addition to their responses to the three dementia-related questions posed. Of the 461 respondents, the vast majority were female (*n* = 418), with 82.4% (*n* = 380) between the age 18–25 years. Nearly two-thirds (*n* = 304) did not have a family member or close friend living with dementia, just over half (57%) had worked with people living with dementia, and 50.8% had no training/education about dementia. Of those that had completed some form of training or education about dementia (*n* = 225), the majority (62%, *n* = 140) had done so outside of the university setting (Table 2).

**Table 2:** Demographics of participants who completed both questionnaires (*n* = 461)

Question	Nursing student total *n* = 395 Frequency (%)	Pharmacy student, total *n* = 66 Frequency (%)
What is your age?		
18–25	316 (80)	64 (97)
26–35	59 (14.9)	2 (3)
36–45	16 (4.1)	0 (0)
46–55	4 (1)	0 (0)
What is your gender?		
Female	374 (94.7)	44 (66.7)
Male/Other	21 (5.3)	22 (33.3)
I have a family member or close friend living with dementia?		
No	264 (66.8)	40 (60.6)
Yes	129 (32.7)	26 (39.4)
Did not respond	2 (0.5)	0 (0)
I work with people living with dementia		
No	147 (37.2)	49 (74.2)
Yes	248 (62.8)	17 (25.8)
I have completed training/education about dementia		
No	175 (44.3)	59 (89.4)
Yes—At University & Outside University	51 (12.9)	0 (0)
Yes—Outside University only	135 (34.2)	5 (7.6)
Yes—At University only	32 (8.1)	2 (3)
Did not respond	2 (0.5)	0 (0)

For analysis purposes male/non-binary has been combined to avoid non-disclosure of participants

Overall, the mean ADQ total score for the nursing students (*n* = 395) demonstrated a significant increase from 79.69 (± 6.08) pretest to 83.59 (± 6.08) posttest (*p* < 0.001). Similarly, a significant increase in mean scores were found for the Hope and Recognition of Personhood subscales (both *p* < 0.001) (Table 3).

**Table 3:** Paired *t*-test (*n* = 395) of pre-game (T_0_) and post-game (T_1_) ADQ scores for nursing students

	Mean (SD)					
	T_0_	T_1_	T	Sig. (2-tailed)^a^	df	Mean gain
ADQ Total score	79.69 (± 6.44)	83.59 (± 6.08)	15.41	*p* < 0.001	394	3.90
ADQ Hope subscale	28.77 (± 3.86)	31.22 (± 4.27)	14.77	*p* < 0.001	394	2.45
ADQ Recognition of Personhood subscale	50.93 (± 4.06)	52.38 (± 3.12)	8.79	*p* < 0.001	394	1.45

*ADQ* Approaches to Dementia Questionnaire, *SD* Standard deviation

^a^For all analyses, a Bonferroni corrected alpha value of *p* ≤ 0.008 indicated statistical significance

Overall, the mean ADQ total score for the pharmacy students (*n* = 66) demonstrated a significant increase from 75.55 (± 5.84) pretest to 79.86 (± 6.70) posttest (*p* < 0.001). As per the nursing students, a significant increase in mean scores were found for the Hope and Recognition of Personhood subscales (both *p* < 0.001) (Table 4).

**Table 4:** Paired *t*-test (*n* = 66) of pre-game (T_0_) and post-game (T_1_) ADQ scores for pharmacy students

	Mean (SD)					
	T_0_	T_1_	T	Sig. (2-tailed)^a^	df	Mean gain
ADQ Total score	75.55 (± 5.84)	79.86 (± 6.70)	8.35	*p* < 0.001	65	4.32
ADQ Hope subscale	26.65 (± 3.35)	29.20 (± 3.98)	7.98	*p* < 0.001	65	2.55
ADQ Recognition of Personhood subscale	48.89 (± 3.63)	50.67 (± 3.71)	5.49	*p* < 0.001	65	1.77

*ADQ* Approaches to Dementia Questionnaire, *SD* Standard deviation

^a^For all analyses, a Bonferroni corrected alpha value of *p* ≤ 0.008 indicated statistical significance

## Discussion

This study demonstrated a significant increase in overall attitudes to dementia following the game-based intervention. Predominantly, participants were female (418 out of 461), and approximately 82% were within the 18–25 age bracket. Notably, a substantial portion of participants lacked personal connections to people living with dementia as they did not have family members with the condition. Additionally, over half reported no formal dementia-specific training, with the majority of those who did receive training obtaining it externally. It’s also noted that a high number of student nurses have worked with people with dementia, but pharmacist students hadn’t. This would appear to indicate that, irrespective of personal experience, the dementia game has the potential to improve nursing and pharmacy student attitudes to dementia.

Among nursing students, a statistically significant improvement in dementia-related attitudes was observed post-gameplay, with average scores increasing from 79.69 to 83.59. This change was statistically significant, reflecting a meaningful advancement in understanding and recognising the personhood of individuals with dementia. Similarly, pharmacy students exhibited notable improvements in their dementia-related scores, rising from an average of 75.55 to 79.86 post-gameplay.

Asynchronous learning, as was a feature of the dementia serious game, while offering flexibility and convenience, comes with its own set of challenges [35, 36]. One significant hurdle is the potential for conflicting schedules and external commitments among learners. This can make it difficult for students to allocate dedicated time for studying and engaging with course materials. Additionally, asynchronous learning requires a high degree of self-discipline and motivation, as there are no fixed class times or immediate interactions with instructors or peers. This lack of real-time engagement can sometimes lead to feelings of isolation or detachment from the learning process [37]. Moreover, without the structure of regular class meetings, students may struggle with time management and procrastination. It is essential for learners to actively manage their own progress and ensure they stay on track with the curriculum. Lastly, technological issues, such as access to reliable internet and compatible devices, can pose barriers to effective asynchronous learning for some individuals [38]. However, like previous studies on serious gaming, the response rate of 505/624 (80.93%) illustrates potential student appetite for participating in gamified modes of learning [27, 28, 39].

While the use of an asynchronous digital serious game proved effective in improving students’ attitudes towards dementia in this study, its application in healthcare education does not guarantee universally successful learning experiences [27, 28].

Caserman and colleagues [40] suggest that there are limited serious games for healthcare education that adhere to rigorous quality standards addressing both the serious and game components. Well-designed serious games seek to cultivate positive emotions, motivating players to persist in their engagement, leading to heightened interest in the gameplay and improved academic performance [41]. A recent meta-analysis [42] on serious game use in education also advocated for understanding learners’ attitudes toward serious game-assisted learning, emphasising the need to design suitable pedagogical strategies catering to diverse learner needs and for educators to develop appropriate serious games, enhancing learning outcomes. Therefore, future evaluations of serious games should also evaluate student perspectives on game entertainment, the usability of the serious game, and student attitudes toward the integration of serious games in education.

## Strengths/limitations

The study exhibits several strengths, including the integration of a co-designed serious game about dementia into nursing and pharmacy curricula at one university in Northern Ireland. Real-world stakeholder involvement in developing the serious game also ensures relevance and applicability. The large sample size of 505 participants with an 80.93% response rate across two healthcare disciplines is also a strength of this study as it enhances the reliability of the findings. This study also does have some limitations, including potential bias in sample demographics, predominantly comprising females in the 18–25 age group. This study did not include a control group, which limits generalisability. Additionally, recruiting a smaller number of pharmacy students posed challenges in statistical analyses, resulting in an uneven distribution between nurses and pharmacy students. Further, this study is limited to two disciplines at one university. Therefore, future work needs to include other professions across multiple universities. Finally, the study does not explore the entertainment value of the serious game, leaving room for future research in exploring the balance between seriousness and engagement.

## Conclusion

Serious games can be an effective means of learning in educational settings. Well-designed serious games lead to heightened interest in the gameplay, which may improve academic performance. Engaged players are more likely to become deeply immersed in the learning experience provided by serious games. The current study demonstrates the impact of utilising a serious digital game on healthcare professional students’ understanding of dementia. Overall, there was a statistically significant increase in overall attitude towards dementia following the game-based intervention. Consequently, digital serious games hold the potential to reach a wide audience and emerge as a fitting tool for enhancing dementia awareness, as showcased in this research.

## Data Availability

The datasets used and/or analysed during the current study are available from the corresponding author on reasonable request.

## Abbreviations

MHLS: Medicine, Health, and Life Sciences
ADQ: Approaches to Dementia Questionnaire
NI: Northern Ireland
UK: United Kingdom
